# Supplementary material for: Embryonic macrophages orchestrate niche cell homeostasis for the establishment of the definitive hematopoietic stem cell pool
Source: Nat Commun. 2025 May 14;16:4428. doi: 10.1038/s41467-025-59059-9 (PMC12078706; doi:10.1038/s41467-025-59059-9)
Supplement: Supplementary file 1 — Supplementary Information [file 41467_2025_59059_MOESM1_ESM.pdf]

Supplementary Information

Embryonic macrophages orchestrate niche cell homeostasis critical for the establishment of the definitive HSC pool.

Gülce Perçin[1&], Konstantin Riege[2], Julia Fröbel[1], Jonas Metz[3], Stephan Culemann[1], Mathias Lesche[4], Susanne Reinhardt[4], Thomas Höfer[3], Steve Hoffmann[2,5], Claudia Waskow[1,5,6&]

[1] Immunology of Aging, Leibniz Institute on Aging - Fritz Lipmann Institute, Jena, Germany.

[2] Computational Biology of Aging, Leibniz Institute on Aging - Fritz Lipmann Institute, Jena, Germany.

[3] Theoretical Systems Biology, German Cancer Research Center, Heidelberg, Germany

[4] DRESDEN-concept Genome Center, c/o CMCB Center for Molecular and Cellular Bioengineering Technology Platform of the TUD Dresden University of Technology, Dresden, Germany.

[5] Institute of Biochemistry and Biophysics, Faculty of Biological Sciences, Friedrich-Schiller-University, Jena, Germany

[6] Department of Medicine III, Faculty of Medicine, TU Dresden, Germany

[&]Corresponding authors

Claudia Waskow

Claudia.waskow@leibniz-fli.de

Gulce Percin

Guelce.Percin@leibniz-fli.de

Supplementary Methods

Bioinformatic analysis: Supplementary Fig.4c (MSC): Paired-end reads in FASTQ files were aligned to the mouse reference genome (GRCm39) using STAR aligner (version 2.7.10a) [1]. Read pairs were first aligned using STAR's default parameters. This initial step resulted in approximately 75% of read pairs being uniquely mapped to the genome. Reads that failed to align were retained for further analysis. To recover reads from the unmapped fraction, alignment parameters were adjusted to allow mapping of shorter sequences. Specifically, the following STAR parameters were used: outFilterScoreMinOverLread=0, outFilterMatchNminOverLread=0, outFilterMatchNmin=20. This step revealed that many initially unmapped read pairs overlapped short genomic regions, causing read 1 to overlap with read 2 in certain cases. To address this, the alignment was repeated with filter criteria that required at least 40% of bases in each read pair to align successfully. For paired-end reads, this threshold corresponds to 80 bases aligned for 100 bp reads. To further optimize the alignment, overlapping read pairs were merged when the overlap was greater than 10 bases (peOverlapNbasesMin=10). The final alignment was performed using the following refined STAR parameters: peOverlapNbasesMin=10, outFilterScoreMinOverLread=0.40, outFilterMatchNminOverLread=0.40. This approach improved the alignment rate, resulting in approximately 80% of read pairs being uniquely mapped to the reference genome. Uniquely mapped read pairs were quantified at the exon level using the featureCounts tool (version 1.6.5) [2]. The quantification was performed based on the Ensembl genome annotation (version 108) [3], with the following parameters: -p -B -T 12 -t exon -g gene_id and thereafter post-processed using rippchen [4]. Ligands and receptors pairs were obtained from Receptor-Ligand database [5] and filtered according to their base expression using a median TPM of 1 as cutoff within samples of Mesenchymal stem cells (MSCs, data processing see above) or macrophages (data processing see main manuscript). Ligands or receptors significantly differentially expressed in adult versus embryonic macrophages were reported and together with their expressed counter parts (receptors, ligands respectively) subjected to hypergeometric tests for over-representation (without background) followed by semantic clustering.

References

1. Dobin, A. *et al.* STAR: ultrafast universal RNA-seq aligner. *Bioinformatics* **29**, 15–21 (2013).
2. Liao, Y., Smyth, G. K. & Shi, W. featureCounts: an efficient general purpose program for assigning sequence reads to genomic features. *Bioinformatics* **30**, 923–930 (2014).
3. Harrison, P. W. *et al.* Ensembl 2024. *Nucleic Acids Res* **52**, D891–D899 (2024).
4. Konstantin Riege. rippchen v0.10.0. Zenodo https://doi.org/10.5281/ZENODO.14187286 (2024).
5. Mende, N. *et al.* Prospective isolation of nonhematopoietic cells of the niche and their differential molecular interactions with HSCs. *Blood* **134**, 1214–1226 (2019).

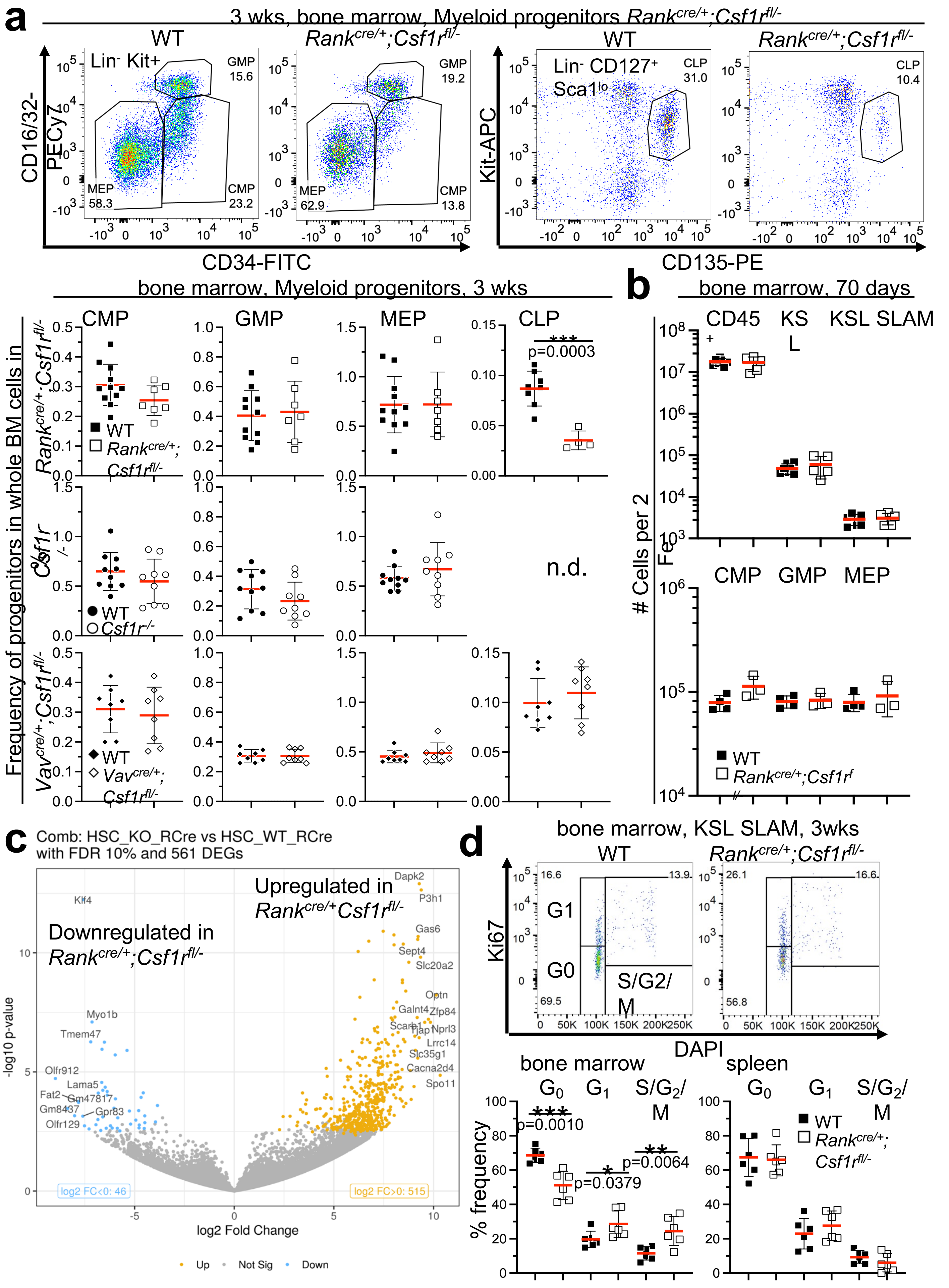

Supplementary Fig. 1 Hematopoietic Stem and Progenitors in 3-week-old and adult *Rank*$^{cre/+}$*;Csf1r*$^{fl/-}$ mice. a Dot plots depict 3-week-old (19-27 day old) *Rank*$^{cre/+}$*;Csf1r*$^{fl/-}$ bone marrow CMP, GMP,MEP and CLP cells in *Rank*$^{cre/+}$*;Csf1r*$^{fl/-}$ and control mice (top). Frequencies of bone marrow progenitor populations in 3-week-old (19-27-day-old) *Rank*$^{cre/+}$*;Csf1r*$^{fl/-}$, *Csf1r*$^{-/-}$ and *Vav*$^{cre/+}$*;Csf1r*$^{fl/-}$ and control mice: common myeloid progenitor (CMP), granulocyte monocyte progenitor (GMP), megakaryocyte-erythroid progenitor (MEP), and common lymphoid progenitor (CLP)(bottom). Myeloid progenitors were identified as Kit+ Sca1- compartment which then further divided into CMP (CD16/32^{-} CD34^{+}), GMP (CD16/32^{+} CD34^{+}) and MEP (CD16/32^{-} CD34^{-}) compartment. Lymphoid progenitors (CLPs) were defined as lineage negative (CD3/CD19/NK1.1/Ter119/CD11b/Gr1/B220 neg), CD127^{+}, Sca1^{lo}, Kit^{+}, CD135^{+} cells. A two-sided unpaired Student's t-test was used. (n=19, 5 biological replicates for *Rank*$^{cre/+}$*;Csf1r*$^{fl/-}$, n=19, 4 biological replicates for *Csf1r*$^{-/-}$,n=16, 3 biological replicates for *Vav*$^{cre/+}$*;Csf1r*$^{fl/-}$). **b** Leukocyte numbers per 2 femura in 70-day-old *Rank*$^{cre/+}$*;Csf1r*$^{fl/-}$ mice and controls. A two-sided unpaired Student's t-test was used. (n=12, 3 biological replicates for HSPCs, n=7, 2 biological replicates for progenitors). **c** Volcano plot depicts 561 genes differentially expressed between HSCs (KSL Slam) from 3-week-old (19-21-day-old) *Rank*$^{cre/+}$*;Csf1r*$^{fl/-}$ and control mice. Upregulated genes in *Rank*$^{cre/+}$*;Csf1r*$^{fl/-}$ HSCs are depicted in yellow, while downregulated genes are depicted in blue. **d** Plots show the frequency of KSL SLAM of indicated genotypes in all cell cycle phases as determined by DAPI and Ki67 staining (top). Graphs show the quantification of G0, G1 and S/G2/M phase of the bone marrow and spleen KSL SLAMs (bottom) from 3-week-old (19-21-day-old) mice. A two-sided unpaired Student's t-test was used. (n=12, 3 biological replicates for bone marrow and spleen).Source data are provided as a Source Data file.

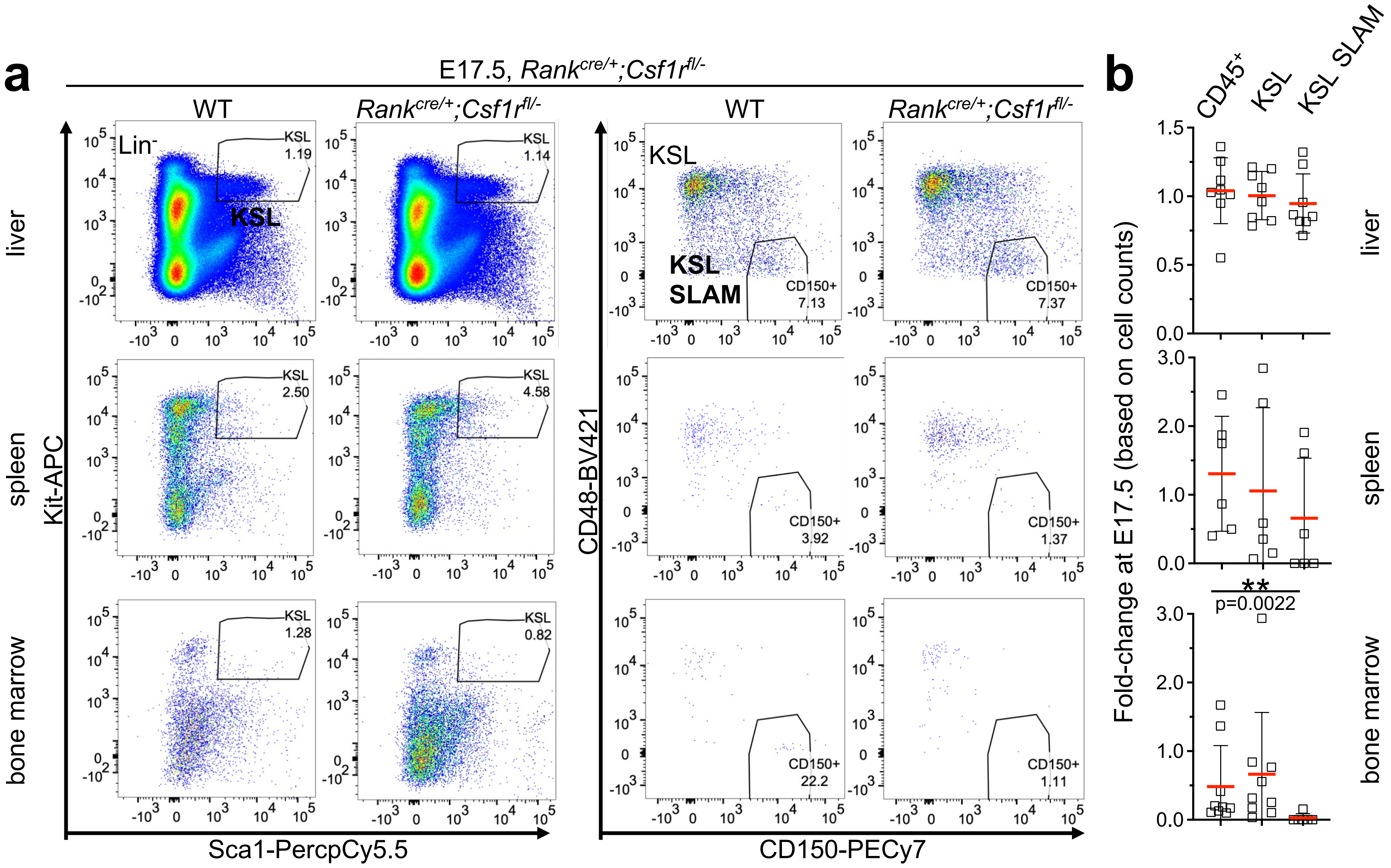

Supplementary Fig. 2 Hematopoietic Stem and Progenitor numbers in E17.5 *Rank$^{cre/+}$;Csf1r$^{fl/-}$* embryos.

a Dot plots depict E17.5 liver, spleen and bone marrow KSL and KSL SLAM cells in *Rank$^{cre/+}$;Csf1r$^{fl/-}$* and control mice. Live singlets were gated on lineage mix (CD3/CD19/NK1.1/Ter119/CD11b/Gr1/B220 neg) followed by Kit^{+} Sca1^{+} (KSL) gating. **b** Plots show fold-changes of leukocytes (CD45), HSPCs (KSL) in the liver, spleen, and bone marrow of E17.5 embryos of indicated genotypes. Fold-change was calculated by dividing the individual hematopoietic cell numbers from *Rank$^{cre/+}$;Csf1r$^{fl/-}$* mice to the experimental average of wildtype values per indicated organ. A Mann–Whitney U test was performed. (n=18, 3 biological replicates for fetal liver, n=21, 3 biological replicates for fetal spleen, n=27, 4 biological replicates for fetal bone marrow). Source data are provided as a Source Data file.

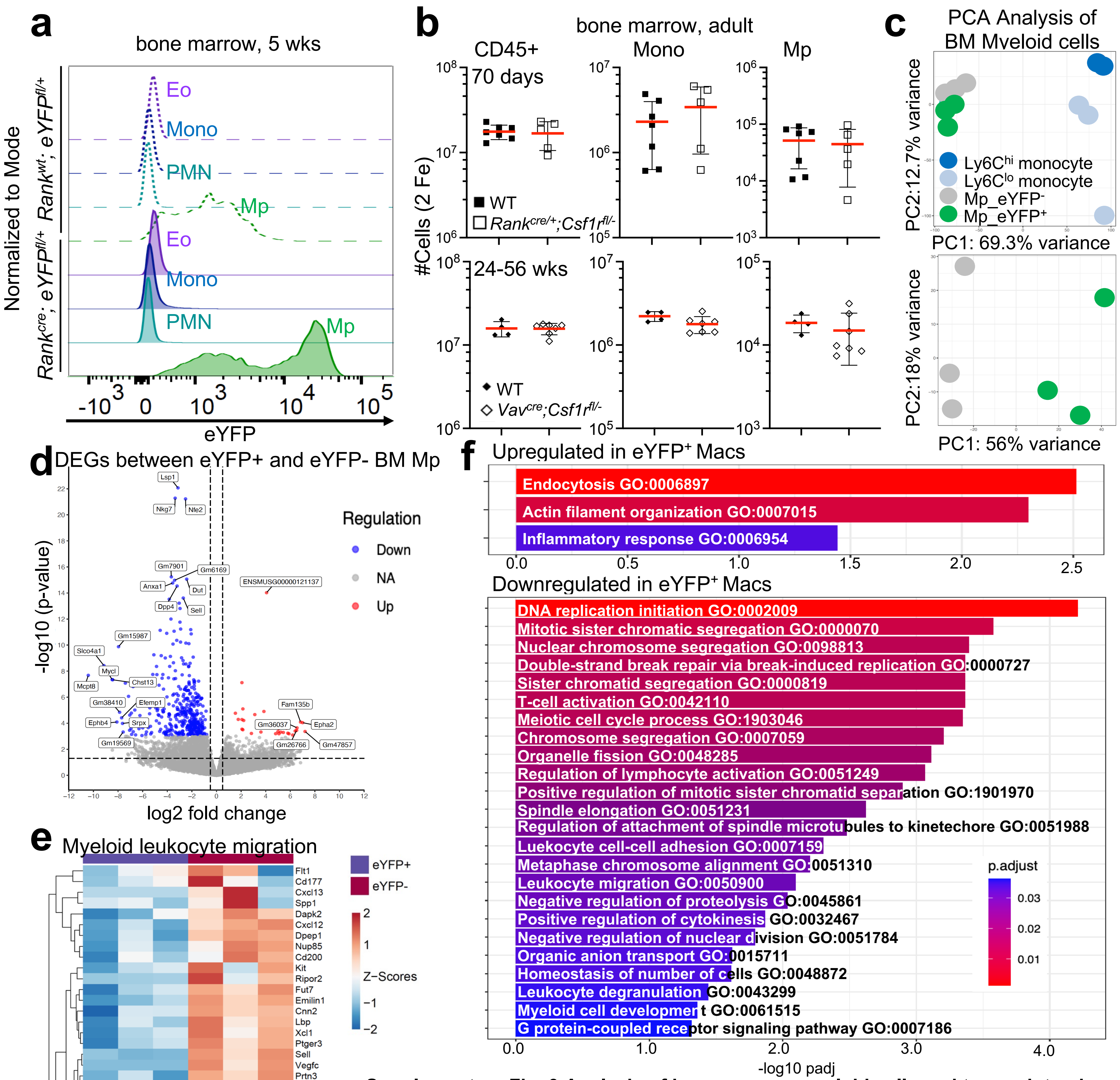

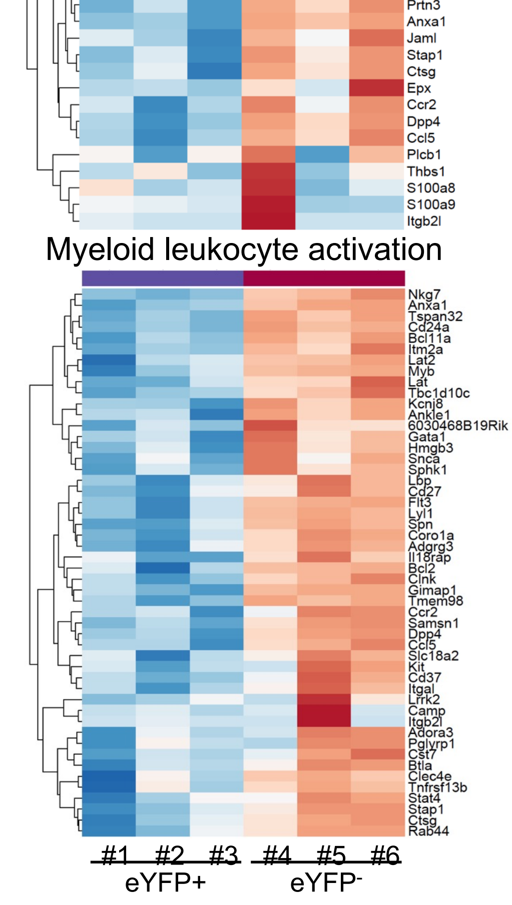

Supplementary Fig. 3 Analysis of bone marrow myeloid cells and transcriptomics between embryonic and adult bone marrow macrophages. a Histograms show eYFP expression in bone marrow myeloid cells in 5-week-old *Rank*$^{cre/+}$*;R26.eYFP*$^{fl/+}$ and control mice. Mp=macrophages, Eo=eosinophils, mono=monocytes, PMN=polymorph nuclear neutrophils. **b** Bone marrow leukocyte (CD45+), monocyte (mono) and macrophage (Mp) numbers in adult *Rank*$^{cre/+}$*;Csf1r*$^{fl/-}$ and control mice (70-day-old, top) and *Vav*$^{cre/+}$*;Csf1r*$^{fl/-}$ and control mice (24-56-week-old, bottom). A two-sided unpaired Student's t-test was used. (n=12, 4 biological replicates for *Rank*$^{cre/+}$*;Csf1r*$^{fl/-}$, n=12, 4 biological replicates for *Vav*$^{cre/+}$*;Csf1r*$^{fl/-}$). **c** Principal Component Analysis (PCA) of the transcriptome of sorted bone marrow Ly6Chi and Ly6Clo monocytes and embryonic eYFP^{+} (Mp_eYFP+) and adult eYFP^{-} (Mp_eYFP-) macrophages from adult (8-week-old) mice (top). PCA of sorted bone marrow embryonic eYFP^{+} and adult eYFP^{-} macrophages only (bottom). **d** Volcano plot depicts 432 significantly differentially expressed genes with Benjamini-Hochberg adjusted p-values below 0.05 (401 downregulated, 31 upregulated) between embryonic (eYFP^{+}) and adult (eYFP^{-}) bone marrow macrophages. Genes that are upregulated in eYFP^{+} macrophages are depicted in red, and downregulated genes are shown in blue. **e** Heatmap showing Z-score normalized expression of genes associated with *myeloid leukocyte migration* (GO:0097529) and *myeloid leukocyte activation* (GO:0002274) sorted bone marrow embryonic eYFP^{+} and adult eYFP^{-} macrophages from adult mice (8-week-old). Colors indicate the Z-score. **f** Plots show biological processes that are enriched in genes up- or down-regulated in embryonic eYFP+ bone marrow macrophages compared to adult eYFP- bone marrow macrophages. Over-representation analysis with a p-adjusted cutoff of 0.05 and subsequent semantic clustering was performed for 401 down-regulated genes using the gene ontology for biological processes from MSigDB, 31 up-regulated respectively using GO annotation from Ensembl. Colors indicate the p-adjusted value. Source data are provided as a Source Data file.

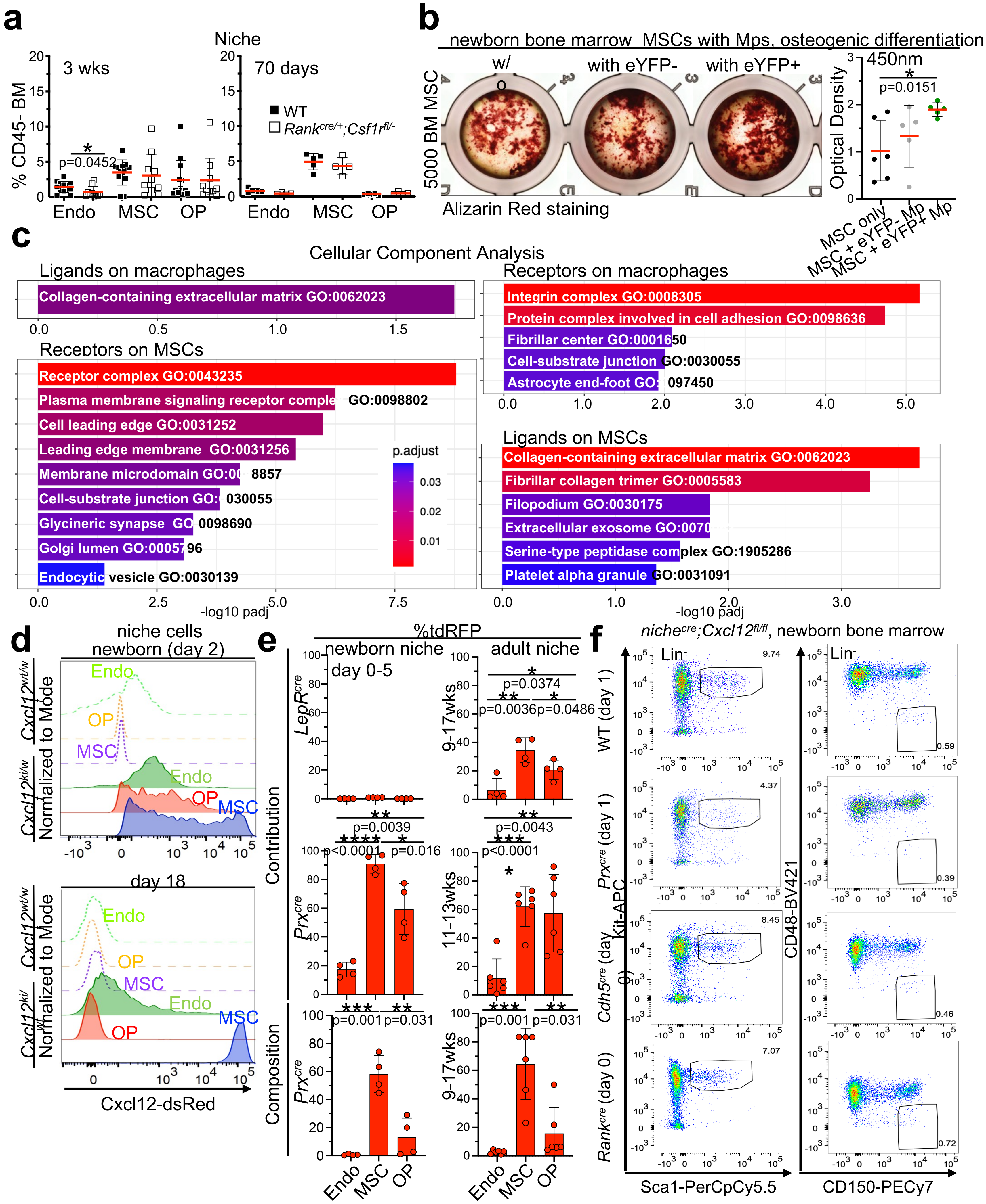

Supplementary Fig. 4: Niche cells in 3-week-old and adult *Rank*$^{cre/+}$*;Csf1r*$^{fl/-}$ mice and analysis of niche cell contribution in neonates. a Frequency of niche cells 3-week-old (top) and 70-day-old (bottom) *Rank*$^{cre/+}$*;Csf1r*$^{fl/-}$ and control mice gated as shown in Fig.4a. A two-sided unpaired Student's t-test was used. (n=23, 5 biological replicates for newborn *Rank*$^{cre/+}$*;Csf1r*$^{fl/-}$, n=9, 3 biological replicates for adult *Rank*$^{cre/+}$*;Csf1r*$^{fl/-}$). **b** Photographs show alizarin red stained osteogenic cultures of sorted MSCs from newborn (8-9-day-old) wild type mice, co-cultured with sorter purified bone marrow macrophages (14 days). Graph shows the quantification of osteogenic differentiation (absorbance at 450 nm). A two-sided unpaired Student's t-test was used. (n=18, 2 biological replicates). **c** Bar plots illustrating enrichment of molecular interactions between embryonic eYFP+ or adult eYFP- bone marrow macrophages with MSCs in cellular component gene sets provided by MSigDB. Ligands being significantly differentially expressed (top), receptors respectively (bottom). Interaction data retrieved from (https://github.com/hoefer-lab/CellInteractionScores). Colors indicate the p-adjusted value. **d** Histograms of CXCL12-dsRed expression in indicated niche cell types in the bone marrow of newborn (2-day-old) and 18-day-old *Cxcl12-dsRed*$^{ki/wt}$ and control mice. **e** Contribution of *Lepr*$^{cre/+}$ (top), or *Prx*$^{cre/+}$ (middle) positive cells to indicated niche cells in neonate (1-5-day-old) *Lepr*$^{cre/+}$*;tdRFP*$^{fl/wt}$ mice and adult (9-17-week-old) mice. A two-sided unpaired Student's t-test was used. (n=4, 2 biological replicates). Composition of tdRFP+ labeled niche cells in neonate *Prx*$^{cre/+}$*;tdRFP*$^{fl/wt}$ (0-5-day-old) and adult mice (bottom). A two-sided unpaired Student's t-test was used. (n=6, 2 biological replicates). **f** Dot plots show bone marrow HSPC analysis of newborn (0-1-day-old) mice of indicated genotypes. Source data are provided as a Source Data file.

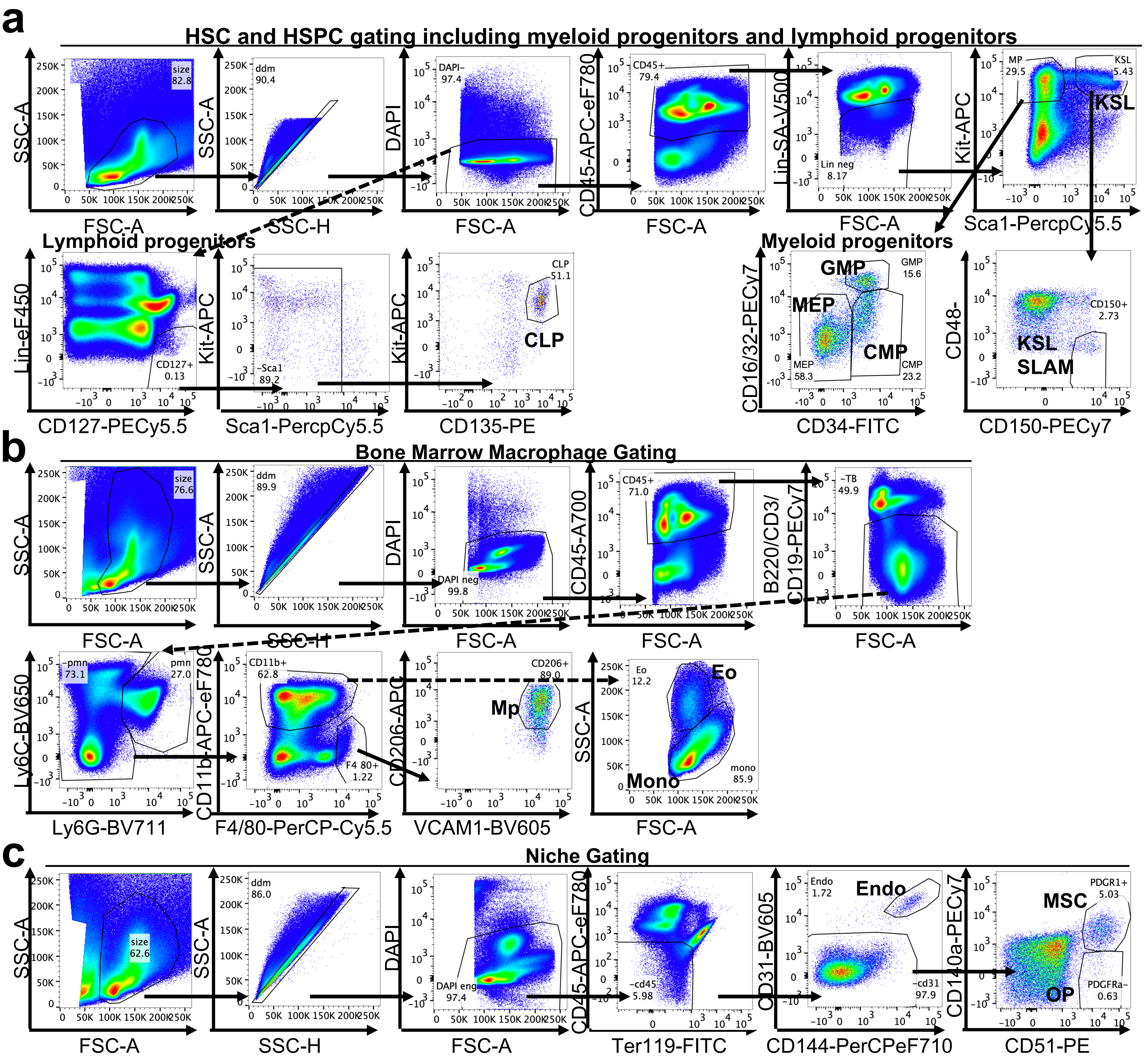

Supplementary Fig. 5: Gating strategy for cell populations described in the paper. a Dot plots depict the gating strategy for 3-week-old bone marrow KSL, KSL SLAM myeloid progenitors: CMP, GMP,MEP and CLP cells in control mice. Dot plots depict bone marrow cells of indicated mice at 3 weeks (18-32-day-old) of age resolved for the expression of indicated antigens. Dapi-negative singlets were gated on lineage negative cells (Lin = CD3 CD19 NK1.1 Ter119 CD11b Gr1 B220) followed by Kit^{+} Sca1^{+} (KSL) gating. KSL cells were further subdivided into CD48^{-} CD150^{+} KSL SLAM cells. Myeloid progenitors were identified as Kit^{+} Sca1^{-} compartment which then further divided into CMP (CD16/32^{-} CD34^{+}), GMP (CD16/32^{+} CD34^{+}) and MEP (CD16/32^{-} CD34^{-}) compartment. Lymphoid progenitors (CLPs) were defined as lineage negative (CD3/CD19/NK1.1/Ter119/CD11b/Gr1/B220 neg), CD127^{+}, Sca1^{lo}, Kit^{+}, CD135^{+} cells. **b** Dot plots show gating for myeloid cells in the bone marrow of *Rank$^{cre/+}$;R26.eYFP$^{fl/+}$* mice. For macrophages, dapi-negative singlets were gated on CD45^{+} cells. B220, CD3, and CD19 postitive cells were further subdivided by Ly6C and Gr1. Gr1^{low} cells were further gated on CD11b vs F4/80. Monocytes and Eosinophils were identified as CD11b^{+} cells separated by scatter characteristics as shown. Macrophages were gated on F4/80^{+} cells and further subdivided by CD206^{+} and VCAM1^{+}. Mp=macrophages, Eo=eosinophils, mono=monocytes, PMN=polymorph nuclear neutrophils. **c** Dot plots display non-hematopoietic niche cells in the bone marrow of newborn (2 days) *Rank$^{cre/+}$;Csf1r$^{fl/-}$* and control mice. From dapi-negative singlets leukocytes (CD45^{-}) and red blood cells (Ter119^{-}) were excluded and endothelial cells (endo) identified by cell surface expression of CD31 and CD144. CD31 negative cells were further subdivided by CD51 and PDGFRa to identify mesenchymal stromal cells, MSC and osteoprogenitors, OP.
