# Supplementary material for: Embryonic macrophages orchestrate niche cell homeostasis for the establishment of the definitive hematopoietic stem cell pool
Source: Nat Commun. 2025 May 14;16:4428. doi: 10.1038/s41467-025-59059-9 (PMC12078706; doi:10.1038/s41467-025-59059-9)
Supplement: Supplementary file 2 — Description of Additional Supplementary Files [file 41467_2025_59059_MOESM2_ESM.pdf]

Description of Additional Supplementary Files

Supplementary Data 1: related to Supplementary Fig.1c (ml)
Significantly differentially expressed genes from a comparison of HSCs from 3-week-old (19-21-day old) *Rank*$^{cre/+}$*;Csf1r*$^{fl/-}$ and control mice.

Supplementary Data 2: related to Fig.1c
Over-representation analysis using significantly differentially expressed genes, both up and down-regulated, in HSCs from 3-week-old (19-21-day old) *Rank*$^{cre/+}$*;Csf1r*$^{fl/-}$ and control mice.

Supplementary Data 3: related to Supplementary Fig. 3f
Over-representation analysis using significantly differentially expressed genes, separate for up and down-regulated, in eYFP^{+} bone marrow macrophages from 8-week-old *Rank*$^{cre/+}$*;R26.eYFP*$^{fl/+}$ mice.

Supplementary Data 4
Differentially expressed receptors or ligands in macrophages (embryonic versus adult) and associated, expressed receptors or ligands on MSCs with median TPM greater than 1.

Supplementary Data 5
List of antibodies used in this study.

Supplementary Data 6
Genotyping information.
