# Supplementary material for: Embryonic macrophages orchestrate niche cell homeostasis for the establishment of the definitive hematopoietic stem cell pool
Source: Nat Commun. 2025 May 14;16:4428. doi: 10.1038/s41467-025-59059-9 (PMC12078706; doi:10.1038/s41467-025-59059-9)
Supplement: Supplementary file 9 — Reporting Summary [file 41467_2025_59059_MOESM9_ESM.pdf]

nature portfolio

Corresponding author(s): Claudia Waskow
Last updated by author(s): Feb 12, 2025

Reporting Summary

Nature Portfolio wishes to improve the reproducibility of the work that we publish. This form provides structure for consistency and transparency in reporting. For further information on Nature Portfolio policies, see our Editorial Policies and the Editorial Policy Checklist.

Statistics

For all statistical analyses, confirm that the following items are present in the figure legend, table legend, main text, or Methods section.

n/a	Confirmed	
☐	☒	The exact sample size (*n*) for each experimental group/condition, given as a discrete number and unit of measurement
☐	☒	A statement on whether measurements were taken from distinct samples or whether the same sample was measured repeatedly
☐	☒	The statistical test(s) used AND whether they are one- or two-sided<br>*Only common tests should be described solely by name; describe more complex techniques in the Methods section.*
☐	☒	A description of all covariates tested
☐	☒	A description of any assumptions or corrections, such as tests of normality and adjustment for multiple comparisons
☐	☒	A full description of the statistical parameters including central tendency (e.g. means) or other basic estimates (e.g. regression coefficient) AND variation (e.g. standard deviation) or associated estimates of uncertainty (e.g. confidence intervals)
☐	☒	For null hypothesis testing, the test statistic (e.g. *F*, *t*, *r*) with confidence intervals, effect sizes, degrees of freedom and *P* value noted<br>*Give P values as exact values whenever suitable.*
☒	☐	For Bayesian analysis, information on the choice of priors and Markov chain Monte Carlo settings
☒	☐	For hierarchical and complex designs, identification of the appropriate level for tests and full reporting of outcomes
☒	☐	Estimates of effect sizes (e.g. Cohen's *d*, Pearson's *r*), indicating how they were calculated

Our web collection on statistics for biologists contains articles on many of the points above.

Software and code

Policy information about availability of computer code

Data collection	BD FACSDiva Software Version 9.0 was used for all flow cytometry data collection
Data analysis	R version 4.1.2 (2021-11-01)<br>Rstudio 2022.07.2+576 FlowJo v9<br>Fiji<br>GraphPad Prism Version 9

For manuscripts utilizing custom algorithms or software that are central to the research but not yet described in published literature, software must be made available to editors and reviewers. We strongly encourage code deposition in a community repository (e.g. GitHub). See the Nature Portfolio guidelines for submitting code & software for further information.

Data

Policy information about availability of data

All manuscripts must include a data availability statement. This statement should provide the following information, where applicable:

- Accession codes, unique identifiers, or web links for publicly available datasets
- A description of any restrictions on data availability
- For clinical datasets or third party data, please ensure that the statement adheres to our policy

All data generated or analyzed during this study are included in this published article (and its supplementary information files). Full descriptions of experimental

procedures and bioinformatic methods can be found in the Method section. Sequencing and processed data generated in this study have been deposited on Gene Expression Omnibus (GEO) under accession numbers GSE265965 for bulk RNA-seq data of sorted HSCs from Rankcre/+;Csf1rfl/- and control BM, GSE265827 for bulk RNA-seq data of murine bone marrow macrophages of embryonic and adult origin, GSE282288 for bulk RNA-seq data of niche cells. Source data are provided as a Source Data file.

Research involving human participants, their data, or biological material

Policy information about studies with human participants or human data. See also policy information about sex, gender (identity/presentation), and sexual orientation and race, ethnicity and racism.

Reporting on sex and gender	Not applicable
Reporting on race, ethnicity, or other socially relevant groupings	Not applicable
Population characteristics	Not applicable
Recruitment	Not applicable
Ethics oversight	Not applicable

Note that full information on the approval of the study protocol must also be provided in the manuscript.

Field-specific reporting

Please select the one below that is the best fit for your research. If you are not sure, read the appropriate sections before making your selection.

[x] Life sciences [ ] Behavioural & social sciences [ ] Ecological, evolutionary & environmental sciences

For a reference copy of the document with all sections, see nature.com/documents/nr-reporting-summary-flat.pdf

Life sciences study design

All studies must disclose on these points even when the disclosure is negative.

Sample size	No sample size calculations or statistical methods were performed to predetermine the experimental sample size.
Data exclusions	No data was excluded from the analysis.
Replication	Multiple independent experiments were performed in each figure. All experiments in replication of the outcome were successful. Number of experiments and the number of mice for each figure could be found in Source Data.
Randomization	There was no randomization. Embryos,newborns and adult mice were assigned to their groups based on their genotypes. Both sexes were used in the study. In transplantation experiments, both sexes were used. Randomization was not needed.
Blinding	Investigators were blinded for the analysis of newborn mice. For adult mice the phenotype is obvious so blinded analysis could not be performed. However, blinding was also not relevant to our study because the phenotype with respect to HSC numbers was not sex-specific.

Reporting for specific materials, systems and methods

We require information from authors about some types of materials, experimental systems and methods used in many studies. Here, indicate whether each material, system or method listed is relevant to your study. If you are not sure if a list item applies to your research, read the appropriate section before selecting a response.

Materials & experimental systems

n/a	Involved in the study	
[ ]	[x]	Antibodies
[x]	[ ]	Eukaryotic cell lines
[x]	[ ]	Palaeontology and archaeology
[ ]	[x]	Animals and other organisms
[x]	[ ]	Clinical data
[x]	[ ]	Dual use research of concern
[x]	[ ]	Plants

Methods

n/a	Involved in the study	
[x]	[ ]	ChIP-seq
[ ]	[x]	Flow cytometry
[x]	[ ]	MRI-based neuroimaging

Antibodies

Antibodies used

Antigen/Dilution/Clone/Fluorochrome/Provider/Cat Number
Sca-1/1:500/D7/PerCP-Cy5.5/eBioscience/45-5981-82
CD150 /1:25/TC15-12F12.2/PECy7/BioLegend /115914
CD48/1:400/HM-48-1/BV421/BD Pharmigen/747718
CD117= KIT /1:400/2B8/APC/eBioscience/17-1171-83
CD45 /1:100/30-F11/Alexa Fluor 700/eBioscience/56-0451-82
CD3/1:1000/2C11, /biotin /eBioscience/13-0031-85
CD19/1:500/eBio 1D3/biotin /eBioscience/13-0193-82
NK1.1/1:2000/PK136/biotin /eBioscience/13-5941-85
Ter119/1:500/Ter119/biotin /eBioscience/13-5921-82
CD11b/1:500/M1/70/biotin /eBioscience/13-0112-85
Gr1/1:800/RB6-8C5/biotin /eBioscience/13-5931-85
B220/1:400/RA3-6B2/biotin /eBioscience/13-0452-82
Streptavidin /1:800//V500/BD Pharmigen/561419
CXCR4=CD184/1:100/L276F12/PE/BioLegend /146505
Ki67/1:300/SolA15/eFluor 660/eBioscience/50-5698
CD45.1 /1:75/A20/PE/eBioscience/12-0453
CD45.2/1:50/104/A700/eBioscience/56-0454-82
CD117= KIT /1:200/2B8/BV421/BD Pharmigen/562609
CD27/1:75/LG.7F9/APC/eBioscience/17-0271-82
CD45/1:200/30-F11/Alexa Fluor 780/eBioscience/47-0451-82
CD31 = PECAM1/1:100/390/BV605/BioLegend /102427
CD34 /1:50/RAM34/FITC/eBioscience/11-0341-81
CD16/32 PE-Cy7/1:25/93/PECy7/eBioscience/25-0161-81
CD3/1:200/17A2/eFluor 450/eBioscience/48-0032-82
CD11b/1:1000/M1/71/eFluor 450/eBioscience/48-0112-82
CD19/1:1000/eBio 1D3/eFluor 450/eBioscience/48-0193-82
Gr1/1:1000/RB6-8C5/eFluor 450/eBioscience/48-5931-82
NK1.1/1:300/PK136/eFluor 450/eBioscience/48-5941-82
Ter119/1:400/Ter119/eFluor 450/eBioscience/48-5921-82
B220/1:150/RA3-6B2/eFluor 450/eBioscience/48-0452-82
CD135 /1:100/A2F10/PE/eBioscience/12-1351-83
CD127/1:50/A7R34/PECy5/eBioscience/15-1271-81
F4/80 /1:200/BM8/PerCP-Cy5.5/eBioscience/45-4801-80
F4/80 /1:500/BM8/bio /eBioscience/13-4801-81
CD3 /1:50/2C11/PECy7/eBioscience/25-0031-82
CD19 /1:400/1D3/PECy7/BD Pharmigen/552854
B220 /1:200/RA3-6B2/PECy7/eBioscience/25-0452-82
CD206 /1:200/C068C2/APC/BioLegend /141708
CD45 /1:150/30-F11/PE/eBioscience/12-0451-83
CD11b /1:600/M1/71/APC-eFlour780/eBioscience/47-0112-82
VCAM1 = CD106/1:200/429 MVCAM-A/BV605/BD Pharmigen/745193
Ly6C /1:200/HK1.4/BV650/BioLegend /128049
Ly6C /1:200/HK1.4/PE/BioLegend /128008
Gr1 /1:200/RB6-8C5/BV711/BioLegend /108443
CD51/1:50/RMV-7/PE/eBioscience/12-0512-81
CD51/1:200/RMV-7/biotin/eBioscience/13-0512-81
VE-Cadherin =CD144/1:100/eBioBV13/PerCP eF710/eBioscience/46-1441-82
CD31 = PECAM1/1:800/390/PECy7/BioLegend /25-0311-81
CD31 = PECAM1/1:100/390/A488/BioLegend /102413
Sca1 /1:200/D7/V500/BD Pharmigen/561228
PDGFRa =CD140a/1:25/APA5/APC/eBioscience/17-1401-81
PDGFRa =CD140a/1:150/APA5/PECy7/eBioscience/25-1401-82
Ter119 /1:200/Ter119/APC-eFlour780/eBioscience/47-5921-82
Streptavidin/1:200//APC/eBioscience/17-4317-82
Goat anti-mouse CD31_polyclonal/1:100/-/-/R&D/AF3628
Donkey anti-goat IgG (H+L)/1:500/-/A488 /ThermoFischer/#A11055
Rabbit anti-mouse CD51_monoclonal/1:100/SC56-07/-/ThermoFischer/MA5-32195
Donkey anti-rabbit IgG (H+L) /1:1000//A647/ThermoFischer/#A31573
CD16/32/1:200/-/-/Invitrogen
Rat Immunoglobulin/1:300/-/-/Jackson Immuno Research

Validation

Antigen/Fluorochrome/Validation
Sca-1/PerCP-Cy5.5/Validated by manufacturer against C57BL/6 bone marrow
CD150 /PECy7/Validated by manufacturer against C57BL/6 bone marrow

CD48/BV421/Validated by manufacturer against blood lymphocytes
CD117= KIT /APC/Validated by manufacturer against C57BL/6 bone marrow
CD45 /Alexa Fluor 700/Validated by manufacturer against C57BL/6 bone marrow
CD3/biotin /Validated by manufacturer against BALB/c splenocytes
CD19/biotin /Validated by manufacturer against BALB/c splenocytes
NK1.1/biotin /Validated by manufacturer against C57BL/6 splenocytes
Ter119/biotin /Validated by manufacturer against BALB/c bone marrow
CD11b/biotin /Validated by manufacturer against BALB/c bone marrow
Gr1/biotin /Validated by manufacturer against mouse bone marrow
B220/biotin /Validated by manufacturer against BALB/c splenocytes
Streptavidin /V500/Validated by manufacturer against BALB/c splenocytes
CXCR4=CD184/PE/Validated by manufacturer against C57BL/6 thymocytes
Ki67/eFluor 660/Validated by manufacturer against C57BL/6 splenocytes
CD45.1 /PE/Validated by manufacturer against SJL splenocytes
CD45.2/A700/Validated by manufacturer against BALB/c splenocytes
CD117= KIT /BV421/Validated by manufacturer against C57BL/6 bone marrow
CD27/APC/Validated by manufacturer against C57BL/6 splenocytes
CD45/Alexa Fluor 780/Validated by manufacturer against C57BL/6 bone marrow
CD31 = PECAM1/BV605/Validated by manufacturer against C57BL/6 splenocytes
CD34 /FITC/Validated by manufacturer against C57BL/6 bone marrow
CD16/32 PE-Cy7/PECy7/Validated by manufacturer against BALB/c splenocytes
CD3/eFluor 450/Validated by manufacturer against mouse splenocytes
CD11b/eFluor 450/Validated by manufacturer against BALB/c bone marrow
CD19/eFluor 450/Validated by manufacturer against mouse splenocytes
Gr1/eFluor 450/Validated by manufacturer against C57BL/6 bone marrow
NK1.1/eFluor 450/Validated by manufacturer against C57BL/6 splenocytes
Ter119/eFluor 450/Validated by manufacturer against Swiss Webster bone marrow
B220/eFluor 450/Validated by manufacturer against C57BL/6 splenocytes
CD135 /PE/Validated by manufacturer against C57BL/6 bone marrow
CD127/PECy5/Validated by manufacturer against C57BL/6 splenocytes
F4/80 /PerCP-Cy5.5/Validated by manufacturer against mouse resident oeritoneal exudate cells
F4/80 /bio /Validated by manufacturer against C57BL/6 splenocytes
CD3 /PECy7/Validated by manufacturer against C57BL/6 splenocytes
CD19 /PECy7/Validated by manufacturer against C57BL/6 splenocytes
B220 /PECy7/Validated by manufacturer against BALB/c splenocytes
CD206 /APC/Validated by manufacturer against BALB/c peritoneal macrophages
CD45 /PE/Validated by manufacturer against C57BL/6 splenocytes
CD11b /APC-eFlour780/Validated by manufacturer against C57BL/6 bone marrow
VCAM1 = CD106/BV605/Validated by manufacturer against BALB/c bone marrow
Ly6C /BV650/Validated by manufacturer against C57BL/6 bone marrow
Ly6C /PE/Validated by manufacturer against C57BL/6 bone marrow
Gr1 /BV711/Validated by manufacturer against C57BL/6 bone marrow
CD51/PE/Validated by manufacturer against C57BL/6 bone marrow
CD51/biotin/Validated by manufacturer against C57BL/6 bone marrow
VE-Cadherin =CD144/PerCP eF710/Validated by manufacturer against bEND-3 cells
CD31 = PECAM1/PECy7/Validated by manufacturer against C57BL/6 splenocytes
CD31 = PECAM1/A488/Validated by manufacturer against C57BL/6 splenocytes
Sca1 /V500/Validated by manufacturer against BALB/c splenocytes
PDGFRa =CD140a/APC/Validated by manufacturer against NIH/3T3 cells
PDGFRa =CD140a/PECy7/Validated by manufacturer against NIH/3T3 cells
Ter119 /APC-eFlour780/Validated by manufacturer against BALB/c bone marrow
Streptavidin/APC/Validated by manufacturer
Goat anti-mouse CD31_polyclonal/-/Validated by manufacturer
Donkey anti-goat IgG (H+L)/A488 /Validated by manufacturer
Rabbit anti-mouse CD51_monoclonal/-/Validated by manufacturer
Donkey anti-rabbit IgG (H+L) /A647/Validated by manufacturer
CD16/32/-/
Rat Immunoglobulin/-/Validated by manufacturer

Animals and other research organisms

Policy information about studies involving animals; ARRIVE guidelines recommended for reporting animal research, and Sex and Gender in Research

Laboratory animals

All animal experiments (Mus musculus) were performed in accordance with German animal welfare legislation and were approved by the relevant authorities: Landesdirektion Dresden and the Thüringer Landesamt für Verbraucherschutz (TLV). All mice were bred and kept under specific pathogen free conditions in separated ventilated cages in the animal facility of the TU Dresden or Leibniz Institute on Aging, Jena providing a 12h/12h light/dark cycle (7am-7pm) at a temperature of 22 ± 2dC and 55 ± 10% humidity (40-75%

tolerance limit). They were kindly provided by: Csf1r- Richard Stanley, Csf1rfl Jeffrey Pollard, Vavcre Thomas Graf, Cxcl12fl, Cxcl12-DsRedki Sean Morrison, Cdh5cre Luisa Iruela-Arispe, Rosa26.tdRfpfl Hans-Jörg Fehling. Rankcre mice were generated by our lab C57BL/6J (B6, #000664), Rosa26.LSL-Yfpfl (#006148), Rosa26.LSL-tdTomatofl (#007909), Leprcre (#032457) and Prxcre (#005584) mice were purchased from the Jackson Laboratory. Csf1r- mice were kept on C3H/HeJ, and all other mouse lines on the C57BL/6J genetic background. Mice were sacrificed by decapitation (newborn mice) or cervical dislocation (adult mice). Rankcre/+;Csf1rfl/- mice were generated by first crossing Rankcre/+ males with Csf1r+/- females. Male Rankcre/+;Csf1r+/- offspring was then bred with Csf1rfl/fl females to obtain Rankcre/+;Csf1rfl/- mice. Vavcre/+;Csf1rfl/- mice were generated following the same breeding strategy. Prxcre/+;Cxcl12fl/fl, Cdh5cre/+;Cxcl12fl/fl, and Rankcre/+;Cxcl12fl/fl mice were produced by crossing cre/+ males (specific to each line) with Cxcl12fl/fl females. Male cre/+;Cxcl12fl/+ offspings were crossed with Cxcl12fl/fl females. Lineage tracers were generated by crossing cre/+ males with Rosa26.LSL-YFPfl/fl, Rosa26.tdRFPfl/fl, or Rosa26.LSL-tdTomatofl/fl females. Genotyping information is provided in Supplementary Data 6.

Wild animals

This study did not involve wild animals.

Reporting on sex

These findings presented here, do not apply to one sex.

Field-collected samples

This study does not involve samples collected from the field.

Ethics oversight

All animal experiments (Mus musculus) were performed in accordance with German animal welfare legislation and were approved by the relevant authorities: Landesdirektion Dresden and the Thüringer Landesamt für Verbraucherschutz (TLV).

Note that full information on the approval of the study protocol must also be provided in the manuscript.

Plants

Seed stocks

This study does not involve plants.

Novel plant genotypes

This study does not involve plants.

Authentication

This study does not involve plants.

Flow Cytometry

Plots

Confirm that:

- [x] The axis labels state the marker and fluorochrome used (e.g. CD4-FITC).
- [x] The axis scales are clearly visible. Include numbers along axes only for bottom left plot of group (a 'group' is an analysis of identical markers).
- [x] All plots are contour plots with outliers or pseudocolor plots.
- [x] A numerical value for number of cells or percentage (with statistics) is provided.

Methodology

Sample preparation

Isolation of hematopoietic and niche cells Embryos and newborn mice: timed pregnancies were performed and the day of vaginal plug was evaluated as 0.5 days post-conception. Aorta-Gonad-Mesonephros (AGM) and fetal livers from E10.5 embryos were digested 30 min at 37°C in PBS/5% FCS containing collagenase type 4 (Worthington, 100µg/ml final,#CAS:9001-12-1) and DNAse I (Sigma-Aldrich, #DN25-1G, 100 µg/ml final). The reaction was stopped by adding 12.5 mM EDTA. Fetal spleen and bone marrow, and newborn liver, spleen, and bone marrow were disintegrated between the frosted end of two glass slides without digestion. All samples were filtered through a 100µm filter mesh. Niche cell preparation for newborn, 3-week and 14-week-old mice: Femurs were prepared39. Briefly, femurs were flushed twice with PBS and digested for 30 minutes at 37°C under agitation. Digestion was stopped with PBS/5% FCS, the suspensions were filtered through a 40 µm filter mesh and continued with staining or differentiation culture. Hematopoietic cell isolation from 3-week-old mice: Bones were crushed, and spleens gently disintegrated between the frosted end of two glass slides. Red blood cell lysis (ACK Lysing Buffer, Gibco #A10492-01) was performed for bone marrow (20 sec) and spleen (40 sec). Lysis was stopped by adding PBS/5% FCS. Blood samples: Blood samples were obtained by retro-orbital bleeding. Red blood cell lysis was performed twice for 5 minutes each. Lysis was stopped by adding PBS/5% FCS.

Flow cytometry Cells were stained and blocked with purified CD16/32 (Invitrogen, clone 93) and rat Immunoglobulin (Jackson Immuno Research, 012-000-002) for 40 minutes on ice65. Macrophage samples are blocked with purified CD16/32 and rat Immunoglobulin) for 30 minutes on ice before staining. All antibodies are listed in Supplementary Data 5. Counting beads (CountBright, ThermoFischer, #C36950) were added to each sample during staining. Live/dead discrimination is done by 0.4 µg/ml of DAPI (Biochemica, A1001,000) right before acquisition.
For sorting, macrophages were enriched using biotinylated anti-F4/80 antibodies and then further incubated with anti-biotin

beads (Miltenyi Biotec #130-090-485). HSC and HSPC were sorted from lineage (CD45, Ter119, CD3, CD4, CD8, Gr1, CD11b)-depleted bone marrow cells (MACS). Before acquisition cells were passed through a 40 µm filter mesh. Cell cycle: Bone marrow and spleen cells were stained for cell surface antigens, fixed using BD Cytofix/Cytoperm Fixation and Permeabilization solution (BD #51-2090KZ), frozen (-80°C) and subsequently stained. Samples were acquired and sorted using BD LSRII, BD LSRFortessa, BD FACSAria III, BD FACSAria Fusion and analyzed with FlowJo Software (TreeStar).

Transplantation Fig.1f: 1000 bone marrow KSL cells (CD45.2) from Rankcre/+;Csf1rfl/- or littermate wildtype controls (21-25-day-old) were sorter purified and transplanted into adult RgW41 (CD45.1) recipients 34. Bone marrow chimerism was evaluated 32 weeks after transplantation.
Fig.2f: 4x105 whole bone marrow cells from newborn Rankcre/+;Csf1rfl/- or littermate controls (2-day-old) were transplanted intravenously into adult RgW41 recipients. Blood PMN chimerism was assessed at 5, 9, 14 and 18 weeks and bone marrow HSC (KSL CD48- CD150+) chimerism was evaluated 18 weeks after transplantation. Fig.3d 1000 bone marrow KSL cells (CD45.2) from wildtype mice (21-25-day-old) were sorter purified and transplanted into adult RgW41 (CD45.1) recipients. Bone marrow chimerism was evaluated 32 weeks after transplantation.

In vivo Homing assay 4x10E5 bone marrow cells from newborn Rankcre/+;Csf1rfl/- or littermate controls were transplanted into the liver of neonate wildtype mice. 16 hours later, bone marrow samples were stained and analyzed.

In vitro Migration assay 750-800 liver KSL cells from newborn Rankcre/+;Csf1rfl/- or littermate wildtype (WT) control mice were sorter purified and dispensed to the upper part of the 24-well transwell plate (VWR 8.0 µm, 24 well) containing DMEM (Gibco, 31966-021) with 10% FCS and 1% P/S. The bottom part of each transwell contained 100 ng/ml of murine CXCL12 (rm-SDF1 Peprotech, #250-20A-10UG) or not. After 2.5 hours of incubation at 37°C, cells from the bottom were collected and analyzed by flow cytometry.

Instrument

Samples were acquired and sorted using BD LSRII, BD LSRFortessa, BD FACSAria III, BD FACSAria Fusion

Software

Flow cytometry data was collected using BDFACSDiva Software and analyzed with FlowJo Software (TreeStar).

Cell population abundance

Purity of the sorted cells were assessed by running 5% of the collected sample, through the same instrument used to sort the cells.

Gating strategy

Gating strategies for all relevant cell populations can be found in the Figures and Supplementary Figures.
For E17.5-adult liver, spleen and bone marrow hematopoietic stem cell populations, live singlets were gated on lineage negative (lineage mix:CD3/CD19/NK1.1/Ter119/CD11b/Gr1/B220 neg), followed by Kit+ Sca1+ (KSL) gating. Myeloid progenitors were identified as Kit+ Sca1- compartment which then further divided into CMP(CD16/32- CD34+), GMP (CD16/32+ CD34+) and MEP (CD16/32- CD34-) compartment. KSL cells were further subdivided into CD48- CD150+) KSL SLAM cells.Lymphoid progenitors were (CLPs) were defined as Lineage negative, CD127+ ,Sca1lo , Kit+ CD135+ cells.
For blood KSL cells, live singlets were gated on CD45+ Kit+ leukocytes. Further this population was divided into Kit+ Sca1+ compartment.
For newborn and adult niche cells, live singlets were gated on CD45 and Ter119 negative cells.Endothelial cells=Endo are further gated by double positivity of CD31 and CD144/Sca1. CD31-/Sca1- cells were further gated for CD51+ expression.,which then divided into PDGFRa+ (Mesenchymal Stromal Cells=MSCs) and PDGFRa- (Osteoprogenitors=OPs) populations.
For macrophages,live singlets were gated on CD45+ cells. B220/CD3/CD19+ cells were excluded. B220/CD3/CD19- cells were further gated on Ly6C vs Gr1. Gr1low cells were further gated on CD11b vs F4/80+. Monocytes and Eosinohils were gated on CD11b+ cells and further identified by side scatter and forward scatter.Macrophages were gated on F4/80+ fraction and further gated on CD206 and VCAM1 and double positive population were identified as macrophages.

☒ Tick this box to confirm that a figure exemplifying the gating strategy is provided in the Supplementary Information.
